# Reference Correlation of the Viscosity of Argon

**DOI:** 10.1007/s10765-025-03603-8

**Published:** 2025-07-10

**Authors:** Sofia G. Sotiriadou, Konstantinos D. Antoniadis, Marc J. Assael, Marcia L. Huber

**Affiliations:** 1 Laboratory of Thermophysical Properties and Environmental Processes, Chemical Engineering Department, Aristotle University, 54636 Thessaloniki, Greece; 2 Chemical Engineering Department, University of Western Macedonia, 50100 Kozani, Greece; 3 Applied Chemicals and Materials Division, National Institute of Standards and Technology, 325 Broadway, Boulder, CO 80305 USA

**Keywords:** Argon, Transport properties, Viscosity

## Abstract

This paper presents a new wide-ranging reference correlation for the viscosity of argon, incorporating recent ab initio dilute-gas calculations and critically evaluated experimental data. The correlation is designed to be used with a high-accuracy Helmholtz equation of state that extends from the triple point (83.8058 K) to 700 K, and at pressures up to 1000 MPa. The estimated uncertainty of the correlation based on comparisons with the best experimental data indicate that the uncertainty for the gas at pressures from zero to 0.1 MPa for temperatures from 202 K to 394 K is 0.076% (at *k* = 2), the uncertainty of the best experimental data, offering a significant improvement over the current reference equation that has an uncertainty in this region of 0.5%. A zero-density correlation based on ab-initio values is incorporated that is valid over a temperature range between 84 K and 10 000 K and has an uncertainty of 0.12% (at the 95% confidence level). The estimated uncertainty for moderate pressures from 1 MPa to 100 MPa is 1% for temperatures from roughly 195 K to 300 K, rising to 2% at 175 K. For the high-pressure region, the estimated uncertainty of the correlation is about 2% for temperatures between 175 K and 308 K at pressures from 100 MPa to 606 MPa. For temperatures from 308 K to 700 K at pressures to 5.2 GPa, the equation has an estimated uncertainty of 10%. The estimated uncertainty in the liquid phase at pressures up to 34 MPa is 3%. The correlation behaves in a physically reasonable manner over the full range of applicability of the EOS, although uncertainties may be higher in regions where data were not available for full validation.

**Supplementary Information:**

The online version contains supplementary material available at 10.1007/s10765-025-03603-8.

## Introduction

Argon is a common fluid that has a wide range of industrial uses. As a result of the passage of the CHIPS Act [1] (Creating Helpful Incentives to Produce Semiconductors) of 2022, there is increased interest in improving the calibration of the flow of gases used in semiconductor processing. In the production of semiconductors, flow meters are often calibrated with one gas, and then calibration coefficients for other gases are determined using gas-property data [2]. Viscosity is one of the properties used in the development of some flow-meter models. Recent advances in quantum-chemical ab initio computations [3–6] allow improvement in the representation of the dilute-gas viscosity of argon. It is our goal to incorporate ab initio results to develop an improved correlation for the viscosity of argon that can not only be used for gas-calibration purposes, but that is valid over the entire fluid range incorporating gas, liquid, and supercritical regions.

The viscosity correlation published in 2004 by Lemmon and Jacobsen [7] is widely used, and is considered a standard, valid over liquid, vapor, and supercritical states. According to Lemmon and Jacobsen [7], the uncertainty.

- For the dilute-gas viscosity (defined in their manuscript as *p* < 1 MPa) is generally within about 0.5%, increasing near the triple point.
- In the range 270 to 300 K at pressures less than 100 MPa, for the non-dilute gas and in the liquid, the uncertainty is as low as 1%.
- In the range 180 to 270 K, the uncertainty is about 2%.
- Below 180 K, and away from the critical region, the uncertainty steadily increases to about 5% at the triple point of the fluid.
- The uncertainty in the critical region is higher.

The correlation of Lemmon and Jacobsen [7] was based on the best available experimental data at the time, and hence was limited by the uncertainty of those measurements. The goal of this work is to develop a new viscosity correlation that will incorporate the new dilute-gas limit ab initio calculations for viscosity, to both extend the temperature range, and to lower the uncertainty in the dilute-gas region. In addition, we will include new critically-assessed literature data that became available after 2004.

In a series of papers published over the last ten years, we reported new reference correlations over extended temperature and pressure ranges for the viscosity of some simple fluids (xenon [8], hydrocarbons (*n-*hexane [9], *n*-heptane,[10], *n*-undecane [11], *n*-hexadecane [12], benzene [13], toluene [14], cyclopentane [15]), alcohols (methanol [16], ethanol [17]), glycols (ethane-1,2-diol [18], propane-1,2-diol [19]) and some refrigerants (R-1234yf and R-1234ze(E) [20], R-134a [21], R-161 [22], R-245fa [23], and R-32 [24]). In this paper, the same methodology adopted in any of the aforementioned papers is extended to developing a new reference correlation for the viscosity of argon.

The analysis we use is based on the best available experimental data. A prerequisite to the analysis is a critical assessment of the experimental data. Here we define two categories of experimental data: primary data, employed in the development of the correlation, and secondary data, used simply for comparison purposes. According to the recommendation adopted by the Subcommittee on Transport Properties (now known as The International Association for Transport Properties) of the International Union of Pure and Applied Chemistry, the primary data are identified by a well-established set of criteria [25]. These criteria have been successfully employed to establish standard reference values for the viscosity and thermal conductivity of fluids over wide ranges of conditions, with uncertainties in the range of 1%. However, in many cases, such a narrow definition unacceptably limits the range of the data representation. Consequently, within the primary data set, it is also necessary to include results that extend over a wide range of conditions, albeit with a poorer accuracy, provided they are consistent with other more accurate data or with theory. In all cases, the accuracy claimed for the final recommended data must reflect the estimated uncertainty in the primary information.

The form of correlation we use expresses the viscosity as a function of temperature and density. Experimental data are generally reported in terms of pressure and temperature and an equation of state (EOS) is needed to obtain corresponding densities. If necessary, we first convert temperatures to ITS-90 [26, 27], then use the Helmholtz EOS published by Tegeler et al. [28] to obtain the density for a given temperature–pressure state point. We also use the critical and triple point associated with this EOS; the critical point and other constants for this EOS are given in Table 1. The uncertainty in density of the EOS is less than 0.02% for pressures up to 12 MPa and temperatures up to 340 K except for the critical region, and less than 0.03% for pressures up to 30 MPa and temperatures between 235 K and 520 K. Elsewhere, the uncertainty in density is generally within 0.2%. The EOS is recommended for use from the melting line to 700 K at pressures up to 1000 MPa, but gives physically reasonable extrapolation behavior up to very high pressures and temperatures [28].

**Table 1:** Critical point and fixed constants for the EOS of Tegeler et al. [28]

Property	Symbol	Units	Value	
Critical temperature	*T*_c_	K		150.687
Critical pressure	*P*_c_	MPa		4.863
Critical density	*ρ*_c_	kg·m^−3^		535.6
Triple-point temperature	*T*_tp_	K		83.8058
Molar mass	*M*	g·mol^−1^		39.948
Molar gas constant	*R*	J·mol^−1^·K^−1^		8.31451

## The Viscosity Correlation

The viscosity *η* can be expressed [8–24, 29–31] as the sum of four independent contributions,

1 $$\eta \,\left( {\rho ,{\rm T}} \right)\,\; = \;\,\eta_{0} {\kern 1pt} \left( {\rm T} \right)\;\, + \;\,\eta_{1} {\kern 1pt} \left( {\rm T} \right)\,\rho \,\; + \;\,{\Delta }\eta \left( {\rho ,{\rm T}} \right)\,\; + \;\,{\Delta }\eta_{{\text{c}}} {\kern 1pt} \left( {\rho ,{\rm T}} \right),$$

where *ρ* is the molar density, *T* is the absolute temperature, and the first term, *η*_0_(*Τ*) = *η*(0,*Τ*), is the contribution to the viscosity in the dilute-gas limit, where only two-body molecular interactions occur. The linear-in-density term, *η*_1_(*Τ*) *ρ*, known as the initial density dependence term, can be separately established using Rainwater-Friend theory [32–34] for the transport properties of moderately dense gases. The critical enhancement term, Δ*η*_c_(*ρ,Τ*), arises from the long-range density fluctuations that occur in a fluid near its critical point, which contribute to divergence of the viscosity at the critical point. This term for viscosity is significant only in the region near the critical point, as shown in Vesovic et al. [35] and Hendl et al. [36]. For CO_2_, Vesovic et al. [35] showed that the enhancement contributes greater than 1% to the viscosity only in the small region bounded by 0.986 < *T*_r_ < 1.019 and 0.642 < *ρ*_r_ < 1.283 (where *T*_r_ and *ρ*_r_ denote the reduced temperature *T*_r_ = *T*/*T*_c_ and reduced density *ρ*_r_ = *ρ*/*ρ*_c_). Since data close to the critical point are unavailable, Δ*η*_c_(*ρ*,*Τ*) will be set to zero in Eq. 1 and not discussed further. The reader should note that theory indicates that the viscosity diverges at the critical point [37], and our model does not have the correct theoretical behavior. Finally, the term Δ*η*(*ρ*,*T*), the residual term, represents the contribution of all other effects to the viscosity of the fluid at elevated densities including many-body collisions, molecular-velocity correlations, and collisional transfer.

The identification of these four separate contributions to the viscosity and to transport properties in general is useful because it is possible to some extent to treat *η*_0_(*Τ*), and *η*_1_(*Τ*) theoretically. In addition, it is possible to derive information about both *η*_0_(*Τ*) and *η*_1_(*Τ*) from experiment. In contrast, there is little theoretical guidance concerning the residual contribution, Δ*η*(*ρ*,*Τ*), and therefore its evaluation is based entirely on an empirical equation obtained by fitting experimental data.

In addition to performing literature searches and using content in previous correlations, we made extensive use of the NIST ThermoData Engine [38] to identify data sources. Table 2 summarizes, to the best of our knowledge, the experimental measurements of the viscosity of argon reported in the literature. Data sources in italics indicate that the particular set was also employed in the development of the correlation of Lemmon and Jacobsen [7]. With few exceptions, we only included in the primary dataset measurements where the technique employed, and the uncertainty of the measurement are specified. Very few data sets specifically call out if the uncertainty is on a *k* = 1 or *k* = 2 basis; we assume *k* = 2 when no information is given. Furthermore, with the exceptions discussed below, we preferred measurements with uncertainty less than 1%. In the remainder of this manuscript all uncertainties are at the *k* = 2 level unless specified otherwise.

**Table 2:** Viscosity measurements of argon

Investigators/reference^a^	Publ Year	Technique^b^	Purity (%)	Uncertainty (%)	No. data	Temperatur range (K)	Pressure range (MPa)
Primary data							
Zhou et al. [39]	2024	VBW	99.999	4.21^2σ^	19	89–200	0.3–5.0
Xiao et al. [5, 40]	2020	2CAP	99.999	0.076^2σ^	42	202–395	0, 0.1
Humberg and Richter [41]	2019	RCyl	99.999	0.2^2σ^	46	253–473	0.1–1
Lin et al. [42]	2014	2CAP	99.9997	0.124^2σ^	14	298–653	0
Berg and Burton [43]	2013	CAP	na	0.06^2σ^	1	298	0
Zhang et al. [44]	2013	2CAP	99.999	0.164^2σ^	17	243–393	0.1
Abramson [45]	2011	RBall	99.995	na	31	293–689	490–5170
Vogel [46–48]	2010	OscDQ	99.999	0.15–0.20	81	291–682	0.05–0.16
Wang et al. [49]	2010	OscD	99.99	2^2σ^	17	300	0.1–4.5
Hurly et al. [50]	2003	GV	99.99	0.3	124	293–373	0.1–3.8
*Evers et al.* [51]	2002	RCyl	99.9996	0.15–0.4	81	293–523	0.09–28
*Wilhelm and Vogel* [52]	2000	VBW	99.998	0.20	160	298–423	0.1–20
Diller and Frederick [53]	1989	PCV	99.99	2	91	292–501	1–57
* Hobley et al.* [54]	1989	CAP	na	0.7	5	301–521	0.1
*Mostert *[55, 56]	1989	VBW	na	na	25	174.7	16–471
* Kestin and Ro* [57]	1982	CAP	na	0.3	5	298–473	0.1
Matthews et al. [58]	1982	CAP	99.9	1	11	118–1598	0.1
Barr et al. [59]	1981	CAP	99.99	1	19	173–1598	0.1
* Kestin et al. *[60]	1978	OscD	99.995	0.1–0.3	9	298–773	0.1
Hongo [61]	1978	OscD	99.994	0.3	52	298–373	0.1–13
*Clifford et al.* [62]	1975	CAP	99.9995	0.1	9	321–1300	0.1
*Haynes* [63]	1973	TorC	99.76	2	167	85–298	0.07–35
*Vermesse and Vidal* [64]	1973	CAP	na	0.5	25	308	12–606
*Rabinovich et al.* [65]	1971	CAP	na	1.2	63	298–523	2–59
*Timrot et al.* [66]	1969	OscD	99.95	0.1	7	300–600	0.1
* Guevara et al.* [67]	1969	CAP	na	0.4	21	1100–2100	0.1
*Gracki et al.* [68]	1969	CAP	99.998	0.2–0.3	44	173–298	0.6–17
* Boon et al. *[69, 70]	1967	CAP	99.98	na	6	83–89	0.07–0.1
* Flynn et al*. [71]	1963	CAP	99.995	0.1	27	194–373	2–18
*De Rocco and Halford* [72]	1958	CAP	na	0.5	20	210–471	0.1
Secondary data							
Borjan et al. [73]	2022	VVHPO	99.998	na	34	313,353	0.1–51
Goodwin et al. [74]	2006	MEMS	na	3^2σ^	43	323–423	7–69
May et al. [75]	2006	2CAP	99.9995	0.168^2σ^	21	202–394	0
* Lukin et al*. [76]	1983	CAP	na	0.3	23	76–293	0.1
* Malbrunot et al.* [77]	1983	AcA	na	na	9	84–97	0.07–0.25
*Abachi et al.* [78]	1980	VBW	na	2	12	83–90	0.06–0.14
*Trappeniers et al*. [79]	1980	VBW	na	2	44	223–323	99–897
Vidal et al. [80]	1980	CAP	na	0.5	7	298	0.1–600
* Kestin and Wakeham* [81]	1979	OscD	99.9	0.2	5	300–473	0.1
*Kestin et al.* [82]	1977	OscD	99.9995	0.1–0.2	8	298–673	0.1
*Kestin and Ro* [83]	1976	OscD	na	0.6	9	298–1273	0.1
Lyusternik and Lavushev [84]	1976	FPor	na	na	49	403–1950	0.02–0.1
*Gough et al.* [85]	1976	CAP	99.8	0.5–1.0	11	120–320	0.1
* Baharudin et al*. [86]	1975	BRIL	na	5	6	85–110	0.1
*Timrot et al.* [87]	1975	OscD	na	1.5	39	292–573	0.1–14.5
Carey et al. [88]	1974	AcA	na	0.1	15	294–298	0.1–14
Casparian and Cole [89]	1974	CAP	99.98	na	4	293–422	0.1
*Hellemans et al*. [90]	1974	OscD	na	0.3	8	298–973	0.1
* Kurin and Golubev* [91]	1974	CAP	99.981	2	99	273–423	9–380
*Maitland and Smith* [92]	1974	CAP	99.995	1	11	295–1533	0.1
*Hellemans et al*. [93]	1973	OscD	99.99	0.1–0.3	6	298–770	0.1
Rakshit et al. [94]	1973	OscD	na	1	4	238–308	0.1
Slyusar et al. [95]	1973	FCyl	na	4	215	83–300	0.08–343
* Kestin et al.* [96]	1972	OscD	99.9995	0.1	8	298–973	0.1
*Kestin et al.* [97]	1972	OscD	99.9995	0.1	7	298.973	0.1
*Kestin et al.* [98]	1971	OscD	na	0.2	40	298	0.1–10
*Dawe and Smith* [99]	1970	CAP	99.995	0.5	15	293–1600	0.1
*Golubev* [100, 101]	1970	CAP	na	na	49	273–473	0.1–48
*Hellemans and Zink* [102]	1970	OscD	na	2	44	104–147	0.5–9.6
* Kalelkar and Kestin* [103]	1970	OscD	na	0.5	9	298–1124	0.1
*Kestin et al*. [104]	1970	OscD	na	0.1	8	298–973	0.1
*Clarke and Smith* [105]	1968	CAP	99.9	0.5	12	114–375	0.1
*De Bock et al.* [106]	1967	TorQ	na	3	19	90	0.1–14
*De Bock et al*. [107]	1967	TorQ	na	3	86	88–140	0.1–20
*DiPippo and Kestin* [108]	1967	OscD	na	0.1	23	297–575	0.03–0.18
*DiPippo et al.* [109]	1967	OscD	na	0.1	10	293, 303	0.1–2.3
Andreev et al. [110]	1966	CAP	99.9	1–3	40	294–923	5–51
* van Itterbeek et al.* [111]	1966	OscD	na	na	10	84, 89	0.1–9.8
*Naugle* [112]	1966	UAtt	99.99	na	4	84–112	0.8
*Naugle et al.* [113]	1966	UAtt	99.99	na	59	86–142	2–15
*Rigby and Smith* [114]	1966	CAP	99.95	0.3	15	293–972	0.1
Chakraborti and Gray [115]	1965	CAP	na	1	1	298	0.1
* Saji and Okuda* [116]	1965	CAP	99.9	na	5	84–87	0.1
*Iwasaki et al*. [117]	1964	OscD	99.997	0.1	14	293–303	0.1–5.3
*Kestin and Nagashima* [118]	1964	OscD	99.997	0.4	20	293–303	0.1–5.2
* Lowry et al*. [119]	1964	TorQ	na	2	20	102, 128	0.1–51
* Reynes and Thodos* [120]	1964	CAP	99.998	na	35	373–423	7–82
Saji and Kobayashi [121]	1964	CAP	99.9	na	5	84–86	0.1
* Forster* [122]	1963	OscD	99.8	na	8	85–114	0.1–0.9
*Iwasaki and Kestin* [123]	1963	OscD	99.997	0.1	14	293–303	0.1–5.3
*Kestin and Whitelaw* [124]	1963	OscD	99.997	0.5	48	296–537	0.1–14
*Filippova and Ishkin* [125]	1961	2CAP	99.8	1.5	52	90–273	3.5–15
Thornton [126]	1960	CAP	99.8	1	1	291	0.1
Filippova and Ishkin [127]	1959	2CAP	99.8	1.5	31	90–273	0.1–15
*Kestin and Leidenfrost* [128]	1959	OscD	99.979	0.05	15	293–298	0.03–3.2
*Makita* [129]	1957	RBall	99.9	na	45	298–423	0.1–78
*Zhdanova* [130]	1957	na	na	na	10	90–149	0.1–4.6
* Bonilla et al*. [131]	1956	CAP	99.9	na	22	273–2073	0.1
Jackson [132]	1956	CAP	99.93	na	1	298	0.1
* Makita* [133]	1955	RBall	97.8	na	30	323–573	0.1–10
*Michels et al.* [134]	1954	CAP	na	na	100	273–348	0.1–200
Rietveld et al. [135]	1953	OscD	na	1	9	72–291	0.01–0.1
*Kiyama and Makita* [136]	1952	RBall	97.8	na	40	323–573	0.1–10
*Johnston and Grilly* [137]	1942	VBW	na	0.5	17	77–296	0.1
*Wobser and Mueller* [138]	1941	FBall	na	1.5	5	293–371	0.1
van Itterbeek and van Paemel [139]	1938	OscD	na	na	6	55–294	0–0.13
*Rudenko and Schubnikow* [140]	1934	CAP	na	1.4	4	84–87	0.1
Trautz and Binikele [141]	1930	CAP	na	na	4	293–473	0.1
*Trautz and Zink* [142]	1930	CAP	99	na	21	567–1100	0.1
Trautz and Ludewigs [143]	1929	CAP	99.8	na	4	288–523	0.1
Ishida [144]	1923	OilD	na	3	1	296	0.1
Rankine [145]	1910	CAP	na	na	1	285	0.1
Tanzler [146]	1906	CAP	na	na	4	273–456	0.1
Schultze [147]	1901	CAP	na	na	6	291–456	0.1

^a^Data sources in italics indicate that the particular set was also employed in the development of the correlation of Lemmon and Jacobsen [7]

^b^2CAP, 2-Capillary; AcA, Acoustic Attenuator; BRIL, Brillouin lines; CAP, Capillary; FBall, Falling Ball; FPor, Flow through Porous media; GV, Greenspan Viscometer; MEMS, Method of Microelectromechanical Systems; OilD, Oil Drop; OscD, Oscillating Disk; OscDQ, Oscillating Disk Quartz viscometer; PCV, Piezoelectric Crystal Viscometer; RBall, Rolling Ball; TorQ, Torsional Quartz, UAtt, Ultrasonic Attenuator; VBW, Vibrating Wire; VVHPO, Variable-Volume High-Pressure Optical view cell; na not available

^2σ^uncertainty either explicitly stated or converted to the 95% confidence level

Hence, in the primary sets we included.

1. All measurements with stated uncertainty equal, or less than, 1% (or 2–2.1% at *k* = 2).
2. We included some measurements with larger uncertainties than 1%, or unspecified uncertainties, in order to extend the primary data set to higher pressures (Diller and Frederick [53], Abramson [45]), or low temperatures (Zhou et al. [39], and Haynes [63]). These were included with weights adjusted so that the fit was not overly influenced by them. Abramson [45] did not specify an uncertainty; we assigned the data an estimated uncertainty of 10% and used the data mainly to guide extrapolations to very high (5 GPa) pressures. In order to improve coverage of the liquid region, we added the measurements of Boon et al. [69] to the primary set.

The following points about data usage should also be made:

- The specific measurements of the group of Kestin [96, 98, 103, 108, 109, 124], performed in 1972 and earlier, with the instrument originally constructed by DiPippo [108, 109], as was pointed out both by Vogel [46] and Maitland et al. [148], are subject to a temperature error, and hence were not included in the primary dataset.
- In cases where measurements are superseded by more recent ones at the same or wider conditions, we report only the latest, e.g. the group of Kestin has previous measurements [81, 82, 93, 97, 104, 117, 118, 123, 128, 149, 150], also the group of Smith [85, 92, 99, 105, 114], and Vogel [47, 48, 151].
- The measurements of Trappeniers et al. [79] were not included as Mostert et al. [55] found at cryogenic temperatures inaccuracies in the measurements, due to the interference between the cooling system and the electrical resistance of the leads.
- In some cases, the authors re-evaluated their earlier work and we retained the most recent publications. The 2010 work of Vogel [46] includes both new measurements and re-analyzed values from his earlier works [47, 48]. Also, Mostert et al. [55] recalculated the values of Van der Gulik and Trappeniers [56] with an additional correction factor and thus is included in the primary set instead of Van der Gulik and Trappeniers [56].

All remaining measurements were considered as secondary as they did not satisfy the aforementioned criteria.

**Fig. 1:**
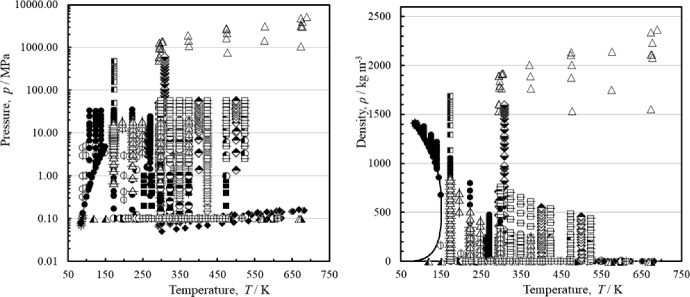
Temperature–pressure and temperature-density ranges of the primary experimental viscosity data for argon, (––) saturation curve. Zhou et al. [39] (), Xiao et al. [5, 40] (×), Humberg and Richter [41] (■), Lin et al. [42] (), Berg and Burton [43] (), Zhang [44] (▲), Abramson [45] (△), Vogel [46] (♦), Wang et al. [49] (□), Hurly et al. [50] (+), Evers et al. [51] (), Wilhelm and Vogel [52] (), Diller and Frederick [53] (), Hobley et al. [54] (), Mostert et al. [55] (), Kestin and Ro [57] (), Matthews et al. [58] (), Barr et al. [59] (), Kestin et al. [60] (), Hongo [61] (), Clifford et al. [62] (), Haynes [63] (●), Vermesse and Vidal [64] (), Rabinovich et al. [65] (), Timrot et al. [66] (○), Gracki et al. [68] (), Boon et al. [69] (∗), Flynn et al. [71] (), de Rocco and Halford [72] ()

Figure 1 shows the temperature–pressure and temperature-density ranges of the primary measurements outlined in Table 2, and the phase boundary. The temperature axis is restricted to 750 K, as measurements in the region above that up to 2100 K are only at atmospheric pressure.

### The Viscosity in the Dilute-Gas Limit

The dilute-gas limit viscosity, *η*_0_(*Τ*) is a function only of temperature and can be analyzed independently of all other contributions in Eq. 1. In 2010, Vogel et al. [4] employed an argon–argon interatomic potential energy curve determined from quantum–mechanical ab initio calculations [3] to calculate the thermophysical properties of argon governed only by two-body interactions. The dilute-gas viscosity was computed from 83.8 K to 10 000 K. The calculated values for the different thermophysical properties were compared with available experimental data and values computed with other argon–argon potentials. An extensive analysis showed that the proposed potential was superior to all previous ones and that the calculated viscosity values were accurate enough to be applied as standard values for the complete temperature range of the calculations.

In 2020, Xiao et al. [5] presented a reference correlation for the dilute-gas viscosity of argon with an uncertainty of about 0.06% (at the *k* = 1 confidence level). Vogel et al. [4] used ab initio computations to compute a reference value of 22.552 μPa·s at 298.15 K for the zero-density gas viscosity of argon. The correlation proposed by Xiao et al. [5] is based on the ab initio computations of Vogel et al. [4], but scaled to match an updated reference value of 22.5666 μPa·s at 298.15 K that is based on the viscosity ratio measurements of May et al. [40].

Very recently, in 2024, Lang et al. [6] determined a new ab initio quantum potential by the inclusion of the two-electron relativistic and leading-order quantum electrodynamics effects. Moreover, the long-range retardation effects were considered to properly describe the dissociation limit.

In this work, it was decided to employ the dilute-gas viscosity correlation proposed by Xiao et al. [5], based on the work of Vogel et al. [4], because the results have been scaled to agree with the most accurate viscosity ratio measurements. This correlation is

2 $$\eta_{0} (T) = \eta_{0} (298.15\;{\text{K}})\exp \left( {\sum\limits_{i = 1}^{12} {a_{i} } \left[ {\ln (T/298.15\;{\text{K}})} \right]^{i} } \right),$$

where *η*_0_(298.15 K) = 22.5666 μPa·s, and the coefficients *a*_*i*_ are shown in Table 3. The computed viscosity values in the dilute-gas limit of Eq. 2 cover a temperature range between 84 K and 10 000 K with an uncertainty of 0.12% (at the 95% confidence level) [5].

**Table 3:** Coefficients *a*_*i*_ (-) of Eq. 2 [5]

*i*	*a*_*i*_	*i*	*a*_*i*_	*i*	*a*_*i*_
1	8.395115 × 10^–1^	5	− 8.881774 × 10^–3^	9	− 2.544782 × 10^–5^
2	− 1.062564 × 10^–1^	6	− 9.613779 × 10^–5^	10	4.398471 × 10^–5^
3	1.065796 × 10^–2^	7	1.404406 × 10^–3^	11	− 9.997908 × 10^–6^
4	1.879809 × 10^–2^	8	− 4.321739 × 10^–4^	12	7.753453 × 10^–7^

**Fig. 2:**
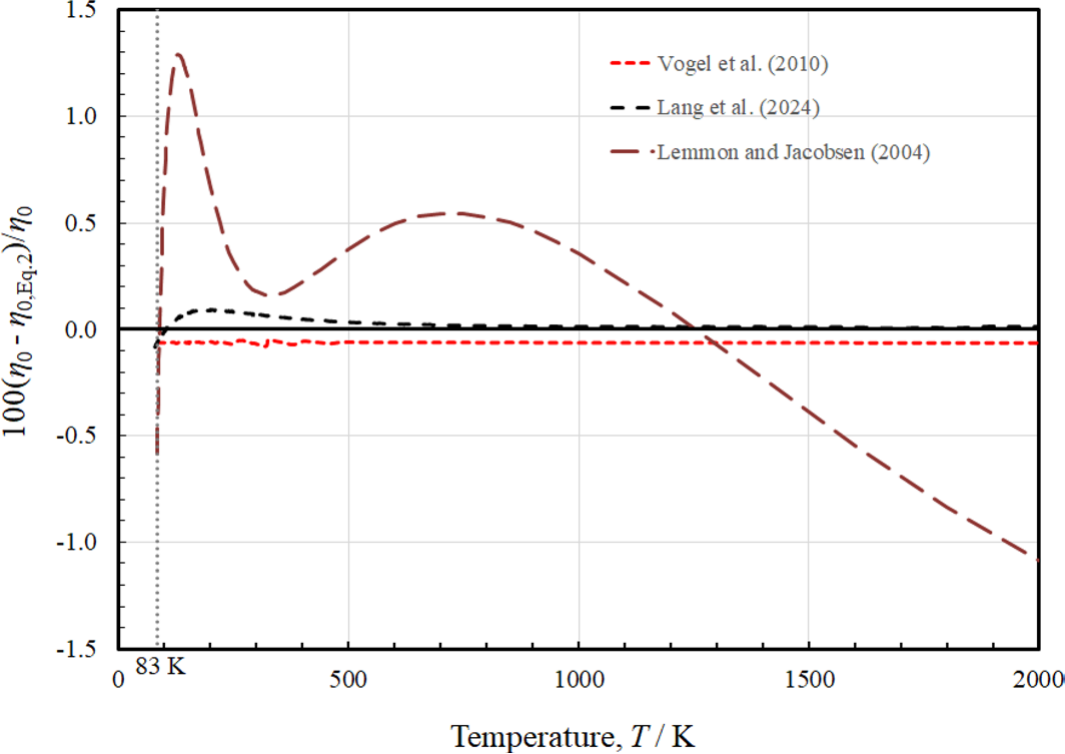
Relative deviations of the dilute-gas viscosity values *η*_0_ from ab-initio calculations and the correlation of Lemmon and Jacobsen [7] from the values calculated by Eq. 2

Figure 2 plotted only up to 2000 K, shows the deviations of the viscosity values calculated from the potentials of Vogel et al. [4], Lang et al. [6], and the correlation of Lemmon and Jacobsen [7] from Eq. 2. The values from Vogel et al. [4] have a small constant offset of about 0.06%, due to the use of a different reference value at 298.15 K, as mentioned earlier. Values from Lang et al. [6] are essentially identical to those from Eq. 2 for temperatures above about 700 K, and have the largest deviation of about 0.09% near 175 K. The correlation of Lemmon and Jacobsen has much larger deviations, with the maximum deviation of 1.3% at about 100 K. As previously mentioned, this correlation was developed only using available experimental data, and incorporating the ab initio results offers significant improvement.

### The Initial-Density Dependence Viscosity Term

We will use theoretical results to guide the development of the initial-density dependence of the viscosity, rather than rely solely on experimental data as was recently done for nitrogen [152]. The temperature dependence of the linear-in-density coefficient of the viscosity *η*_1_(*T*) in Eq. 1 is very large at subcritical temperatures and must be considered to obtain an accurate representation of the behavior of the viscosity in the vapor phase. It changes sign from positive to negative as the temperature decreases. Therefore, the viscosity along an isotherm should first decrease in the vapor phase and subsequently increase with increasing density [153]. Vogel et al*.* [154] have shown that fluids exhibit the same general behavior of the initial density dependence of viscosity, which can also be expressed by means of the second viscosity virial coefficient *B*_*η*_(*T*), as

3 $$\eta_{1} ({ T}) = \eta_{0} ({ T}){ B}_{\eta } ({ T}).$$

The second viscosity virial coefficient can be obtained according to the theory of Rainwater and Friend [32, 33] as a function of a reduced second viscosity virial coefficient, ${ B}_{\eta }^{*} ({ T}^{*} )$, as

4 $${ B}_{\eta } ({T}) = { B}_{\eta }^{*} ({T}^{*} ){N_{A}}_{{\text{}}} \,\sigma^{3} ,$$

where *N*_A_ is the Avogadro constant, *σ* is an intermolecular potential distance parameter, and T* is a scaled temperature that will depend on the intermolecular potential.

${ B}_{\eta }^{*} ({ T}^{*} )$ depends on what potential is used. Vogel et al. [153] presented an expression for ${B}_{\eta }^{*} ({T}^{*} )$ based on a Lennard–Jones potential that has been widely used for a variety of fluids including for example, aromatics [13, 14] and alkanes [9–11]. Najafi et al. [155] used potentials that are more accurate than the Lennard–Jones, specifically that of that of Aziz [156] and Boyes [157] and gave correlations for ${ B}_{\eta }^{*} ({ T}^{*} )$. We will adopt the Najafi et al. [155] correlation based up the Aziz potential [156] as it gives slightly better performance at 300 K [155] than the correlation based on the Boyes potential, and is in better agreement with experimental values than the correlation of Vogel et al. [153]. The Najafi et al. [155] correlation is recommended for use for densities up to 2 mol/L. It is expressed as

5 $${ B}_{\eta }^{*} ({ T}^{*} ) = \sum\limits_{\iota = 0}^{6} {c_{i} \left( {T^{*} } \right)^{ - i} } ,$$

with coefficients *c*_*i*_ given in Table 4, and T* = *T*/(*ε*/k_B_) is a scaled temperature, with parameters *ε*/*k*_B_ = 143.235 K and *σ* = 0.33501 nm [155].

**Table 4:** Coefficients *c*_*i*_ for Eq. 5 [155]

*i*	*c*_*i*_
0	− 0.2571
1	3.033
2	1.144
3	− 5.586
4	3.089
5	− 0.8824
6	− 0.03856

**Fig. 3:**
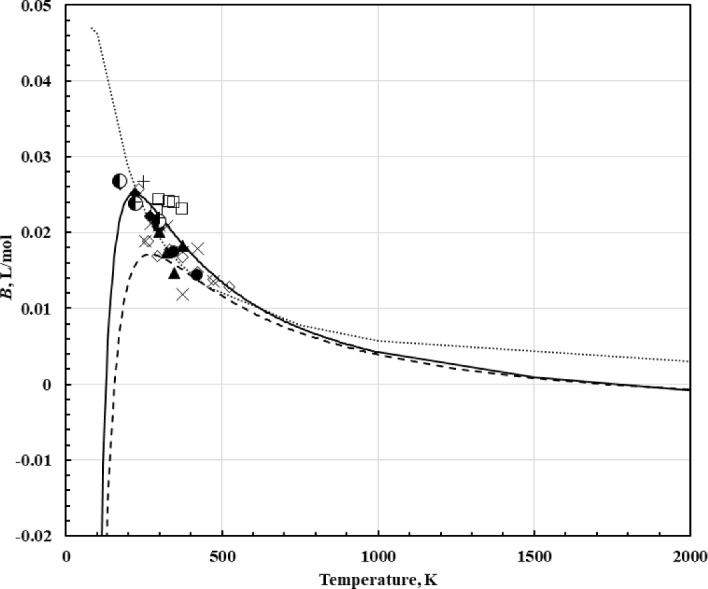
The second viscosity virial coefficient of argon, *B*, as a function of temperature. Correlation of Lemmon and Jacobsen [7] dotted line, Correlation of Vogel et al. [153] dashed line, Correlation of Najafi et al. [155] solid line, Humberg and Richter [41] (×), Hurly et al. [50] (□), Evers et al. [51] (◊), Wilhelm and Vogel [52] (●), Hongo [61] (▲), Haynes [63] (◆), Gracki et al. [68] (), Flynn et al. [71] (+)

Figure 3 shows experimentally-derived values of *B*_*η*_ along with values computed from the correlation of Vogel et al. [153] (incorporating Lennard–Jones parameters recommended by Bich [34]), values computed from the correlation given by Najafi et al. [155] based on the Aziz potential [156], and values derived from the correlation of Lemmon and Jacobsen [7]. The correlation of Lemmon and Jacobsen [7] did not incorporate any theory and is based solely on experimental data; since there were no data at high temperatures the correlation deviates from theory as the temperature increases. It also deviates from theory at very low temperatures, again in a region where experimental data were unavailable. The correlation of Vogel appears to have a lower peak than the experimental data and it is shifted slightly.

### The Viscosity Residual Term

The residual viscosity term Δ*η*(*ρ*,*T*), represents the contribution of all other effects to the viscosity of the fluid at elevated densities including many-body collisions, molecular-velocity correlations, and collisional transfer. Because there is little theoretical guidance concerning this term, its evaluation is based entirely on experimentally obtained data.

The procedure adopted during this analysis used symbolic regression software [158] to fit the primary data to obtain the residual viscosity correlation Δ*η*(*ρ*,*T*). The functional form is not known at the start of the regression process; symbolic regression is used to determine not only the coefficients but also the functional form of the correlation. Symbolic regression is a type of genetic programming that allows the exploration of arbitrary functional forms to regress data. The functional form is obtained by use of a set of operators, parameters, and variables as building blocks. In the present work we restricted the operators to the set (+ , − ,*,/) and the operands (constant, *T*_r_, *ρ*_r_), with *T*_r_ = *T/T*_c_ and *ρ*_r_ = *ρ*/*ρ*_c_. In addition, we adopted a form suggested from the hard-sphere model employed by Assael et al. [159] Δ*η*(*ρ*_r_,*T*_r_) = (*ρ*_r_^2/3^*T*_r_^1/2^)*F*(*ρ*_r_,*T*_r_), where the symbolic regression method was used to determine the functional form for *F*(*ρ*_r_,*T*_r_). For this task, the dilute-gas limit and the initial density dependence terms were calculated for each experimental point (employing Eqs. 2–5) and subtracted from the experimental viscosity to obtain the residual term. We increased the weights on the data as necessary to ensure the residual contribution was near zero for densities less than 2 mol⋅L^−1^ to retain the theoretical values. The final equation obtained was

6 $${\Delta }\eta (\rho ,{\rm T}) = \left( {\rho_{{\text{r}}}^{{2/3}} T_{{\text{r}}}^{{1/2}} } \right)\left\{ {f_{1} \rho_{{\text{r}}} + \frac{{f_{2} \rho_{{\text{r}}}^{2} }}{{T_{{\text{r}}} }} + \frac{{(f_{1} \rho_{{\text{r}}} - \rho_{{\text{r}}}^{{2}} )}}{{T_{{\text{r}}}^{{5}} }} + \frac{{(\rho_{{\text{r}}}^{{}} - f_{3} \rho_{{\text{r}}}^{5} )}}{{(\rho_{{{\text{r}}_{{}} }} - f_{4} - T_{{\text{r}}} )}} - f_{5} } \right\}.$$

**Fig. 4:**
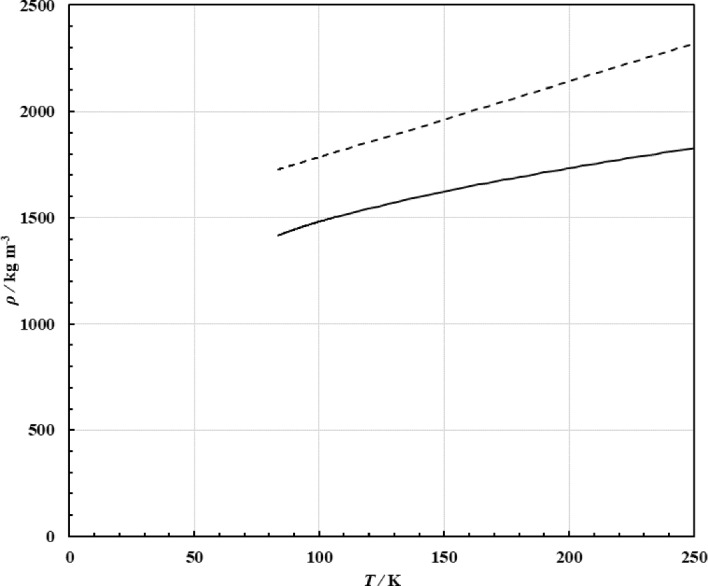
The melting line for argon and location of poles of Eq. 6. Melting line from Tegeler et al. [28] solid line, line of poles from Eq. 6 dashed line

The coefficients are given in Table 5, and Δ*η* is in μPa·s. A parameter file suitable for use with the NIST REFPROP [160] program is included in the supplemental information that gives the full correlation Eqs. 1–6. When using symbolic regression programs, we have noticed that the resulting correlation often has mathematical poles. This is true for the correlation here as well, as there are discontinuities when the denominator of Eq. 6 is zero. For integration into software that may encounter evaluation beyond physically meaningful conditions, we recommend users check that the region *T*_r_ = *ρ*_r_—*f*_4_ is avoided to ensure discontinuities will not be encountered and cause numerical instabilities. For argon, we know the location of the melting line, and the line of poles in Eq. 6 is well into the solid region as shown in Fig. 4. The melting line is that given by Tegeler et al. [28]. An explicit range of applicability is not given, but it is said to behave reasonably up to ~ 750 K and 6000 MPa.

**Table 5:** Coefficients *f*_*i*_ for Eq. 6

*i*	*f*_*i*_
1	3.62648753859904
2	6.655428299399591
3	0.397511608257391
4	2.6697983930209
5	0.0472018570860789

### Comparison with Data

The final correlation model consists of Eqs. 1–6 with the critical enhancement term set to zero. Table 6 summarizes comparisons of the primary data with the present correlation, while Table 7 gives comparisons of the secondary data. Comparisons with the correlation of Lemmon and Jacobsen [7] are also given. We use the following expressions for the percent deviation (PCT), average absolute relative deviation (AARD) and BIAS

7 $${\text{PCT}}_{i} = \frac{{100\;\Delta \eta_{i} }}{{\eta_{i} }} = \frac{{100(\eta_{\exp ,i} - \eta_{{\text{calc,i}}} )}}{{\eta_{{{\text{calc}},i}} }},$$

8 $${\text{AARD = }}\left( {\sum\limits_{i = 1}^{n} {\left| {{\text{PCT}}_{i} } \right|} } \right)/n,$$

9 $${\text{BIAS = }}\left( {\sum\limits_{i = 1}^{n} {{\text{PCT}}_{i} } } \right)/n,$$

where *n* is the number of data points, *η*_exp_ is the experimental value of the viscosity and *η*_calc_ is the value calculated from the correlation. The maximum deviation (positive or negative) is also given.

**Table 6:** Evaluation of the argon viscosity correlation for the primary data

Investigators/references	Year Publ	AARD (%)	BIAS (%)	MAX (%)	AARD (%)	BIAS (%)	MAX (%)
		Present work			Lemmon and Jacobsen [7]		
Zhou et al. [39]	2024	1.79	0.56	3.86	1.15	− 0.05	2.55
Xiao et al. [5, 40]	2010	0.01	0.00	− 0.03	0.28	− 0.28	− 0.62
Humberg and Richter [41]	2019	0.05	− 0.04	− 0.13	0.21	− 0.21	− 0.40
Lin et al. [42]	2014	0.07	− 0.07	− 0.13	0.41	− 0.41	− 0.54
Berg and Burton [43]	2013	0.06	− 0.06	− 0.06	0.23	− 0.23	− 0.23
Zhang et al. [44]	2013	0.07	− 0.07	− 0.09	0.27	− 0.27	− 0.40
Abramson [45]	2011	3.13	− 1.21	9.73	21.26	− 20.05	− 55.61
Vogel [46]	2010	0.05	− 0.05	− 0.18	0.38	− 0.38	− 0.67
Wang et al. [49]	2010	0.31	− 0.02	0.73	0.36	0.08	0.87
Hurly et al. [50]	2003	0.26	0.26	0.34	0.33	0.33	0.56
Evers et al. [51]	2002	0.30	− 0.17	− 2.34	0.23	− 0.09	− 1.92
Wilhelm and Vogel [52]	2000	0.22	− 0.18	− 0.68	0.12	− 0.01	− 0.43
Mostert et al. [55, 56]	1989	0.77	0.45	2.48	1.16	− 0.70	− 2.92
Diller and Frederick [53]	1989	1.30	1.04	4.27	1.43	1.28	3.92
Hobley et al. [54]	1989	0.07	0.06	0.19	0.19	− 0.19	− 0.22
Kestin and Ro [57]	1982	0.81	0.81	1.20	0.60	0.59	0.95
Matthews et al. [58]	1982	0.53	0.11	− 1.59	0.48	− 0.36	− 1.06
Barr et al. [59]	1981	0.49	0.04	− 1.98	0.44	− 0.33	− 2.96
Kestin et al. [60]	1978	0..60	0.60	1.13	0.31	0.20	0.95
Hongo [61]	1978	0.31	0.18	0.86	0.40	0.34	1.39
Clifford et al. [62]	1975	0.37	− 0.05	− 0.78	0.50	− 0.34	− 1.09
Haynes [63]	1973	0.76	0.41	4.83	0.93	0.21	5.72
Vermesse and Vidal [64]	1973	1.09	0.68	2.98	2.44	2.27	7.44
Rabinovich et al. [65]	1971	0.69	− 0.65	− 1.55	0.60	− 0.37	− 1.54
Timrot et al. [66]	1969	0.88	0.88	2.08	0.60	0.57	1.58
Guevara et al. [67]	1969	0.55	0.07	− 0.97	0.59	0.59	0.97
Gracki et al. [68]	1969	0.44	− 0.09	− 1.61	1.10	− 1.09	− 3.22
Boon et al. [69]	1967	0.48	− 0.05	− 1.00	0.48	0.0	− 0.90
Flynn et al. [71]	1963	0.49	0.15	1.94	0.24	0.12	1.12
De Rocco and Halford [72]	1958	0.49	− 0.40	− 0.90	0.65	− 0.63	− 1.22
Total		0.55	0.10		1.11	− 0.42

**Table 7:** Evaluation of the argon viscosity correlation for the secondary data

Investigators/references	Year Publ	AARD (%)	BIAS (%)	MAX (%)	AARD (%)	BIAS (%)	MAX (%)
		Present work			Lemmon and Jacobsen [7]		
Borjan et al. [73]	2022	0.51	− 0.51	− 0.85	0.22	− 0.07	− 0.70
Goodwin et al. [74]	2006	2.66	0.33	8.07	2.60	0.77	8.49
May et al. [75]	2006	0.05	0.05	0.07	0.23	− 0.23	− 0.58
Lukin et al. [76]	1983	1.39	1.20	4.28	0.75	0.33	4.31
Malbrunot et al. [77]	1983	2.51	2.51	4.55	2.75	2.75	5.29
Abachi et al. [78]	1980	2.79	2.79	6.75	2.87	2.87	6.82
Trappeniers et al. [79]	1980	5.05	4.15	13.38	5.27	3.24	− 14.11
Vidal et al. [80]	1980	2.58	− 1.68	− 4.34	2.56	− 0.04	− 4.49
Kestin and Wakeham [81]	1979	0.80	0.80	1.09	0.59	0.59	0.84
Kestin et al. [82]	1977	0.73	0.73	1.06	0.42	0.37	0.81
Kestin and Ro [83]	1976	0.47	0.47	0.64	0.25	0.22	0.46
Lyusternik and Lavushev [84]	1976	0.91	− 0.78	− 1.69	0.85	− 0.76	− 1.95
Gough et al. [85]	1976	0.36	0.36	0.73	0.38	− 0.33	− 1.00
Baharudin et al. [86]	1975	20.59	− 2.57	59.88	20.45	− 1.70	62.20
Timrot et al. [87]	1975	0.35	0.24	1.28	0.39	0.34	1.13
Carey et al. [88]	1974	0.49	0.39	1.42	0.69	0.65	1.70
Casparian and Cole [89]	1974	0.68	0.68	1.45	0.58	0.50	1.26
Hellemans et al. [90]	1974	0.52	0.44	1.24	0.44	0.05	0.90
Kurin and Golubev [91]	1974	1.50	− 0.43	− 8.28	1.25	0.63	6.77
Maitland and Smith [92]	1974	0.53	− 0.09	− 1.13	0.46	− 0.30	− 0.99
Hellemans et al. [93]	1973	0.66	0.66	1.10	0.33	0.29	0.77
Rakshit et al. [94]	1973	1.51	1.51	4.04	1.32	1.25	3.65
Slyusar et al. [95]	1973	1.63	0.42	17.47	1.31	0.93	15.82
Kestin et al. [96]	1972	0.59	0.56	1.07	0.41	0.17	0.73
Kestin et al. [97]	1972	0.54	0.54	1.01	0.30	0.17	0.67
Kestin et al. [98]	1971	0.14	0.12	0.39	0.28	0.28	0.83
Dawe and Smith [99]	1970	0.65	− 0.32	− 1.21	0.65	− 0.48	− 0.98
Golubev [100, 101]	1970	4.09	3.93	19.64	4.43	4.21	19.65
Hellemans and Zink [102]	1970	7.19	− 6.85	− 33.58	6.96	− 6.23	− 33.49
Kalelkar and Kestin [103]	1970	0.38	0.20	0.73	0.43	− 0.16	− 0.67
Kestin et al. [104]	1970	0.43	0.40	0.80	0.31	0.01	0.59
Clarke and Smith [105]	1968	0.25	0.25	0.51	0.52	− 0.41	− 1.80
De Bock et al. [106]	1967	3.13	3.13	5.69	2.18	2.18	5.13
De Bock et al. [107]	1967	4.73	3.91	20.19	4.72	3.54	20.92
DiPippo and Kestin [108]	1967	0.67	0.67	1.14	0.44	0.42	0.72
DiPippo et al. [109]	1967	0.10	0.10	0.21	0.13	0.11	0.27
Andreev et al. [110]	1966	1.42	− 1.40	− 3.21	1.77	− 1.57	− 4.08
van Itterbeek et al. [111]	1966	1.04	0.95	3.98	1.03	0.08	2.88
Naugle [112]	1966	1.37	− 1.37	− 2.78	1.05	− 1.05	− 1.42
Naugle et al. [113]	1966	9.39	− 9.39	− 17.28	9.81	− 9.81	− 17.39
Rigby and Smith [114]	1966	1.67	− 1.67	− 3.25	2.10	− 2.10	− 3.65
Chakraborti and Gray [115]	1965	0.20	− 0.20	− 0.20	0.37	− 0.37	− 0.37
Saji and Okuda [116]	1965	0.43	0.31	0.79	1.30	1.19	2.99
Iwasaki et al. [117]	1964	0.12	− 0.05	− 0.26	0.15	0.09	0.42
Kestin and Nagashima [118]	1964	0.10	0.08	0.34	0.23	0.23	0.64
Lowry et al. [119]	1964	6.90	− 6.84	− 15.79	8.25	− 8.25	− 15.30
Reynes and Thodos [120]	1964	3.43	3.39	6.07	3.59	3.57	6.22
Saji and Kobayashi [121]	1964	0.44	0.33	0.80	0.45	0.36	0.84
Forster [122]	1963	2.80	2.80	5.63	3.61	3.61	7.01
Iwasaki and Kestin [123]	1963	0.12	− 0.05	− 0.26	0.15	0.09	0.42
Kestin and Whitelaw [124]	1963	1.51	1.45	3.06	1.65	1.62	3.48
Filippova and Ishkin [125]	1961	10.64	1.89	52.90	9.80	0.91	45.04
Thornton [126]	1960	0.17	− 0.17	− 0.17	0.35	− 0.35	− 0.35
Filippova and Ishkin [127]	1959	7.14	3.99	− 16.41	6.40	3.22	− 16.36
Kestin and Leidenfrost [128]	1959	0.09	0.01	0.23	0.07	0.01	0.21
Makita [129]	1957	0.99	− 0.59	− 3.72	0.83	− 0.13	− 3.51
Zhdanova [130]	1957	8.22	4.27	21.25	8.69	5.41	22.08
Bonilla et al. [131]	1956	4.74	− 4.72	− 9.74	4.67	− 4.67	− 8.67
Jackson [132]	1956	0.29	− 0.29	− 0.29	0.46	− 0.46	− 0.46
Makita [133]	1955	1.81	0.54	5.68	2.07	0.62	6.26
Michels et al. [134]	1954	0.61	− 0.56	− 1.46	0.26	− 0.11	− 1.54
Rietveld et al. [135]	1953	3.14	1.19	7.38	3.17	0.86	9.11
Kiyama and Makita [136]	1952	1.66	0.33	6.14	1.90	0.39	6.73
Johnston and Grilly [137]	1942	1.32	1.32	6.51	0.72	0.50	6.55
Wobser and Mueller [138]	1941	0.10	0.01	0.18	0.17	− 0.16	− 0.29
van Itterbeek and van Paemel [139]	1938	4.51	4.08	5.42	6.80	6.32	13.23
Rudenko and Schubnikow [140]	1934	2.73	− 2.73	− 3.22	2.70	− 2.70	− 3.18
Trautz and Binikele [141]	1930	1.26	− 1.26	− 1.80	1.53	− 1.53	− 2.20
Trautz and Zink [142]	1930	3.24	− 3.24	− 4.98	3.68	− 3.68	− 5.20
Trautz and Ludewigs [143]	1929	1.12	− 1.12	− 1.91	1.40	− 1.40	− 2.31
Ishida [144]	1923	1.59	− 1.59	− 1.59	1.76	− 1.76	− 1.76
Rankine [145]	1910	0.42	− 0.42	− 0.42	0.61	− 0.61	− 0.61
Tanzler [146]	1906	1.42	1.42	1.84	1.20	1.20	1.53
Schultze [147]	1901	0.53	0.53	1.09	0.31	0.31	0.78

**Fig. 5:**
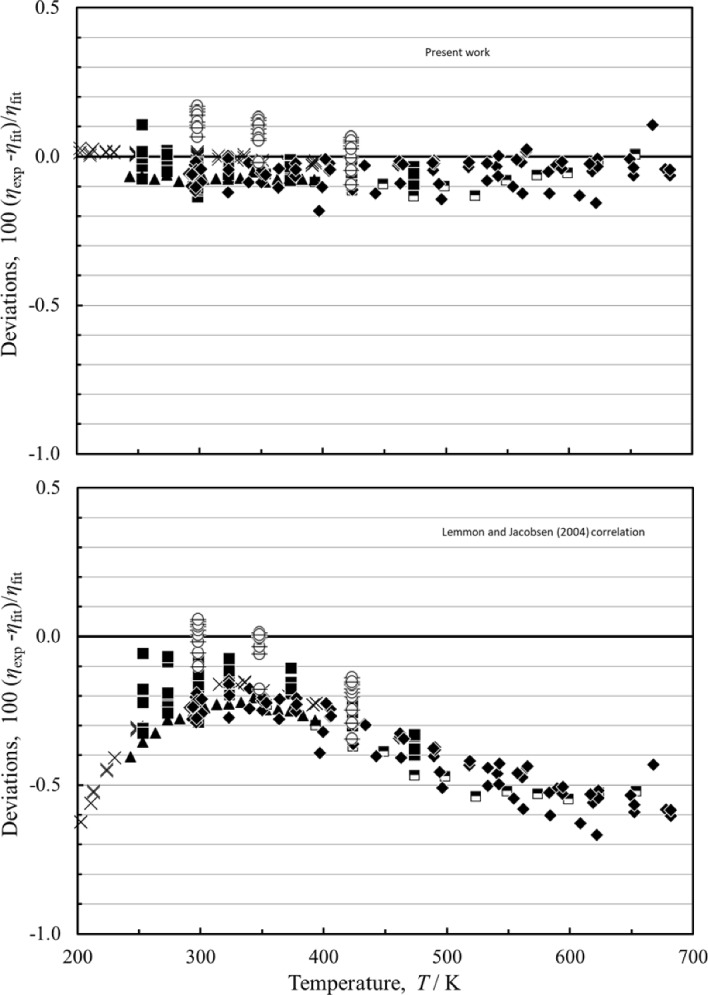
Percentage deviations of selected data at low pressures (*p* < 1 MPa) calculated by the present model and the model of Lemmon and Jacobsen [7]. Xiao et al. [5, 40] ( ×), Humberg and Richter [41] (■), Lin et al. [42] (), Berg and Burton [43] (), Zhang [44] (▲), Vogel [46] (♦), Wilhelm and Vogel [52] ()

One of the primary drivers for this work is to improve upon the correlation of Lemmon and Jacobsen [7] by incorporating developments in theory. This was done by incorporating Eqs. 2–5 for densities below about 2 mol⋅liter^−1^ (80 kg⋅m^−3^). Although we have identified a primary data set, the data were not fit to determine coefficients for this region since the behavior is fixed by Eqs.2–5. Figure 5 shows comparisons with the most reliable low-uncertainty measurements at pressures up to 1 MPa for the present correlation, Eqs. 1–6, and for the Lemmon and Jacobsen [7] correlation. The Lemmon and Jacobsen results are generally within about 0.5%, as claimed. The present correlation gives improved results in this region. Comparisons with the re-analyzed data of May [40] presented in Xiao et al. [5], that cover temperatures from 202 K to 394 K at pressures of zero and 0.1 MPa, show the correlation represents the data to within their stated uncertainty, 0.076% at *k* = 2. For temperatures above 394 K, comparisons with the data of Vogel et al. [46] show agreement to within the uncertainty of the experimental data, which Vogel conservatively reported as 0.15–0.2% with the highest uncertainty at the highest temperature. The Vogel et al. [46] data were calibrated with a reference value of 22.552 μPa s, that is slightly lower than the reference value used in this work, 22.5666 μPa s, and as indicated in Fig. 5 exhibit a small systematic deviation. As noted by Xiao et al. [5], the data of Lin et al. [42] show very good agreement up to about 393 K but have larger deviations as the temperature increases. The zero-density correlation, Eq. 2, [5] incorporated into this work has an estimated uncertainty of 0.12 (at *k* = 2) for the entire range from 83.8 K to 10,000 K.

**Fig. 6:**
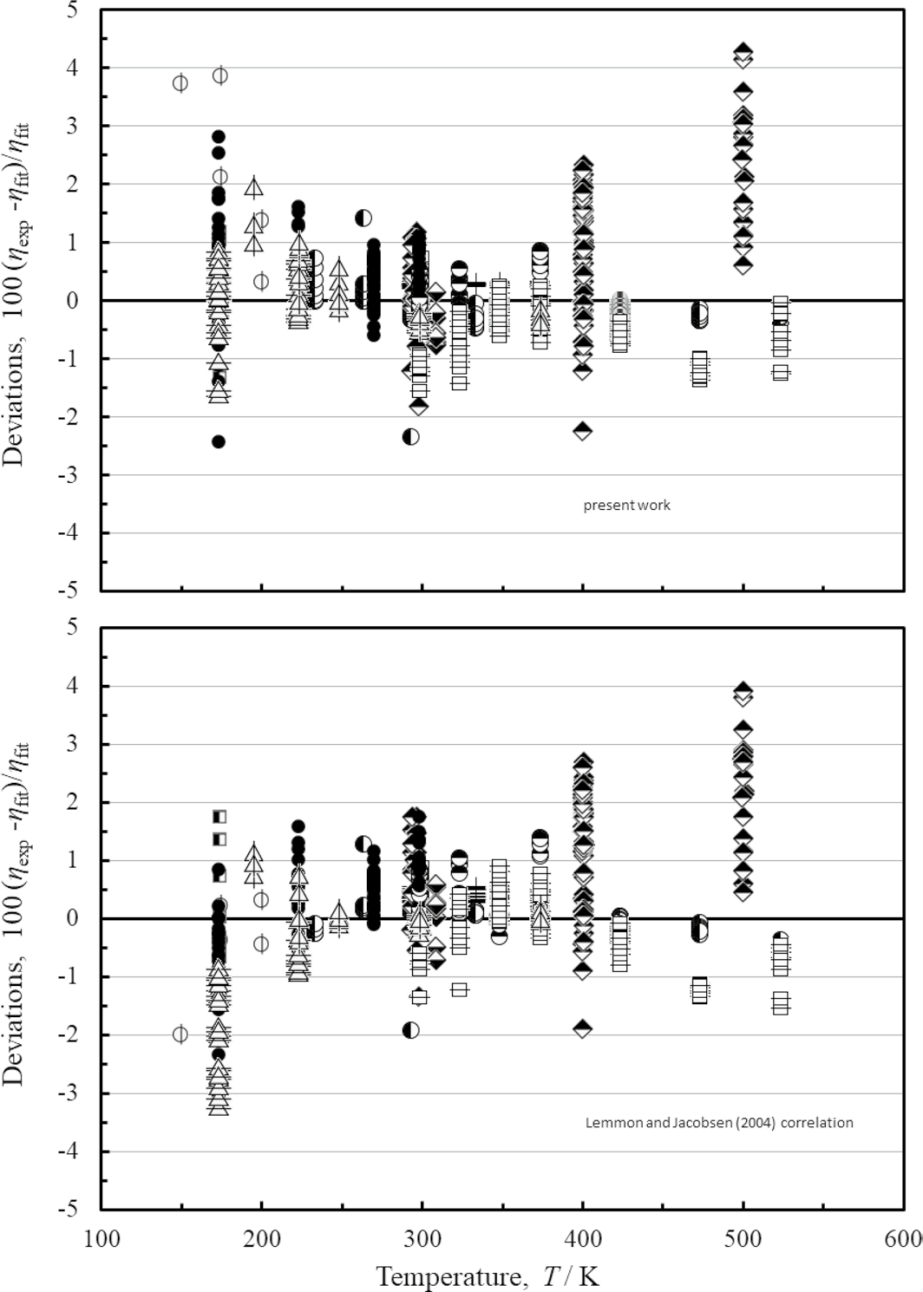
Percentage deviations of the primary data pressures between 1 and 100 MPa calculated by the present model and the model of Lemmon and Jacobsen [7]. Zhou et al. [39] (), Wang et al. [49] (□), Hurly et al. [50] (+), Evers et al. [51] (), Wilhelm and Vogel [52] (), Diller and Frederick [53] (), Mostert et al. [55] (), Hongo [61] (), Haynes [63] (●), Vermesse and Vidal [64] (), Rabinovich et al. [65] (), Gracki et al. [68] (), (Flynn et al. [71] ()

For the mid-range pressure region (1 MPa < *p* < 100 MPa), Fig. 6 indicates that the results of the Lemmon and Jacobsen [7] model and the present model are very similar. Lemmon and Jacobsen [7] state an uncertainty of 1% for the temperature range from 270 to 300 K for pressures between 1 and 100 MPa, and that is the same found for the present correlation. For the temperature range of 180 K to 270 K and pressures between 1 and 100 MPa, the Lemmon and Jacobsen correlation [7] claims a 2% uncertainty, the present correlation gives 1% for this region. However, the Lemmon and Jacobsen [7] manuscript did not identify a primary data set and used all available data for comparisons, including some with larger deviations, such as Trappeniers et al. [79], that were excluded from our primary data set. When the Lemmon and Jacobsen [7] correlation is compared with our primary data set, it also has a 1% uncertainty for the temperature range of 180 K to 270 K and pressures between 1 and 100 MPa. Below 180 K, the most significant data sets useful for comparisons are Gracki et al. [68], Haynes [63], and Mostert et al. [55, 56], all of which have measurements that do not go below 173 K. For this region the uncertainties rise to about 3%. Below 173 K, there are not enough data at pressures between 1 and 100 MPa to do significant comparisons; only Zhou et al. [39] has one point at 150 K and 3.5 MPa that indicate that the uncertainty is at least 4% in this region. Additional high-quality measurements at temperatures below 173 K and at pressures up to 100 MPa could help improve the correlation in this region.

**Fig. 7:**
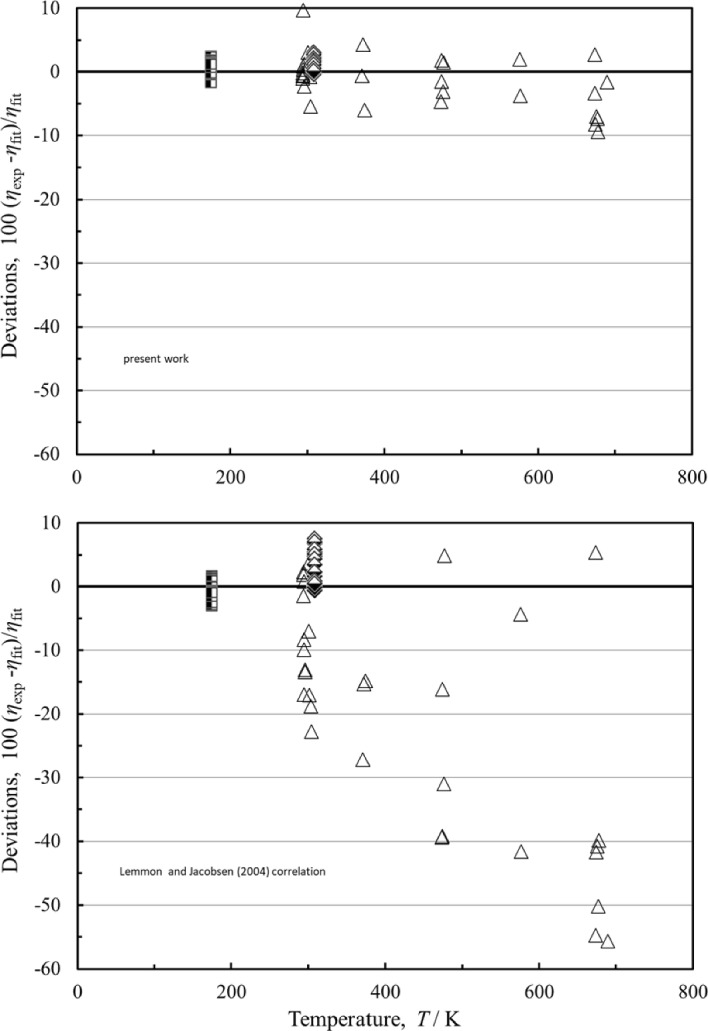
Percentage deviations of the primary data pressures between 100 and 6000 MPa calculated by the present model and the model of Lemmon and Jacobsen [7]. Abramson [45] (△), Mostert et al. [55] (),Vermesse and Vidal [64] ()

Figure 7 shows the deviations for both models as a function of temperature for the high-pressure region above 100 MPa. In this region the present correlation has improved performance over the Lemmon and Jacobsen correlation [7] due to the inclusion of the data of Abramson et al. [45] that were unavailable to Lemmon and Jacobsen [7]; these measurements extend to very high pressures (5.17 GPa). Unfortunately Abramson et al. [45] did not give uncertainties for the measurements, and we have assigned an uncertainty of 10% based primarily on the observed scatter in the data and performance of the instrument on other fluids [152, 161]. Based on the measurements of Mostert et al. [55] and Vermesse and Vidal [64], the estimated uncertainty of the correlation is about 2% for temperatures between 175 K and 308 K at pressures from 100 MPa to 606 MPa. Until additional data are available, we can only claim 10% uncertainty for temperatures above 308 K and high pressures.

**Fig. 8:**
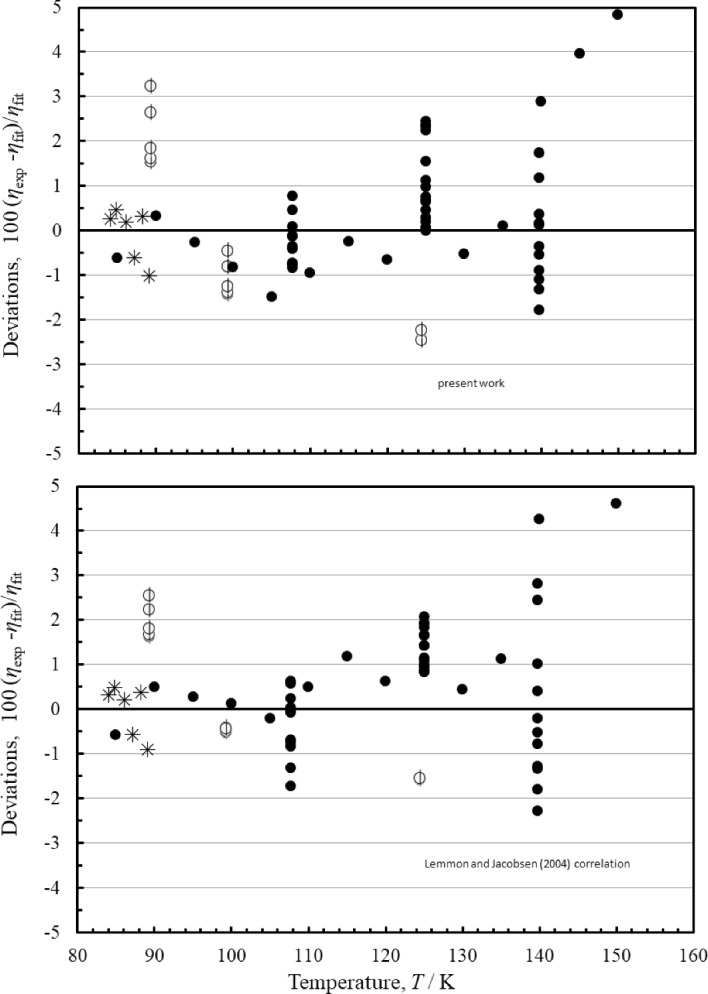
Percentage deviations of the primary data in the liquid phase at pressures up to 34 MPa calculated by the present model and the model of Lemmon and Jacobsen [7]. Zhou et al. [39] (), Haynes [63] (●), Boon et al. [69] (∗)

Figure 8 shows deviations for both correlations for liquid-phase measurements. These extend up to 34 MPa. The performance of both correlations is similar; the estimated uncertainty in this region based on comparisons with the experimental data is about 3%.

**Fig. 9:**
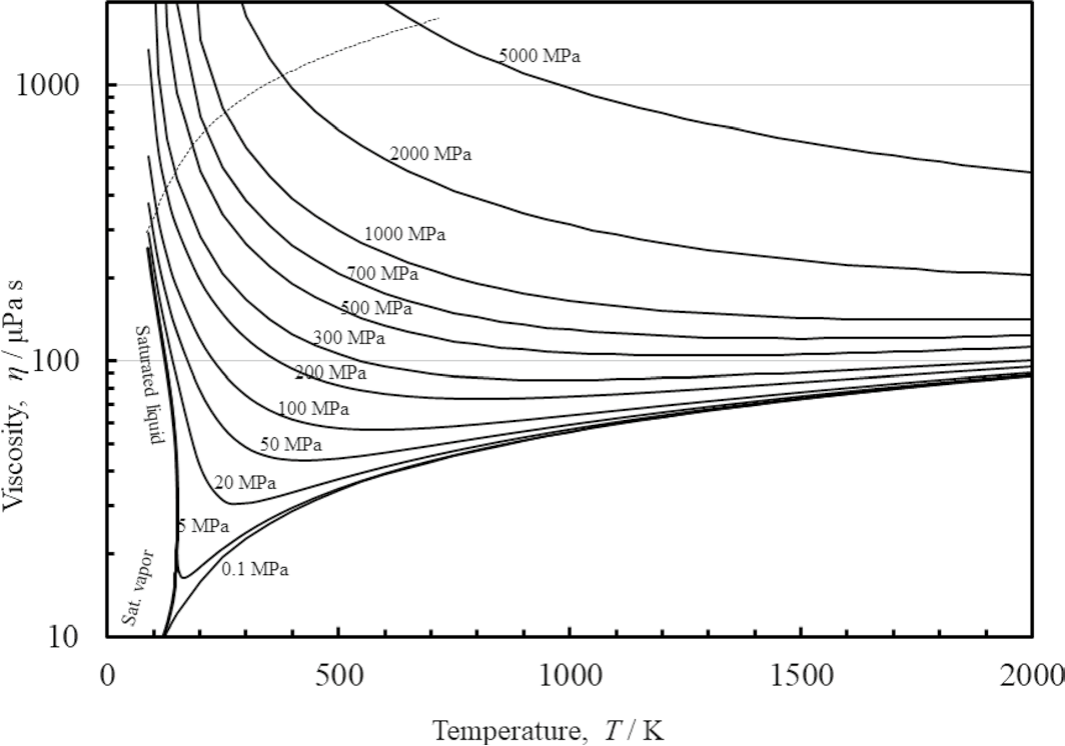
Viscosity of argon as a function of the temperature for different pressures. The extrapolated melting line of Tegeler et al. [28] is indicated by the dotted line

Finally, Fig. 9 shows a plot of the viscosity of argon as a function of the temperature for different pressures. The plot demonstrates the reasonable extrapolation behavior at pressures up to 5 GPa and temperatures to 2000 K, that exceed the limits of the current EOS of Tegeler et al. [28] (700 K and 1 GPa). It is difficult to assign an uncertainty at conditions where there are no experimental data, so we can only state that the behavior is physically reasonable (no discontinuities, and the isobars on the temperature-viscosity plot do not cross). The extrapolated melting line of Tegeler et al. [28] is indicated by the dotted line, and the correlation does not have unphysical behavior such as mathematical poles within the fluid region. As noted earlier, the correlation does exhibit unphysical behavior outside of this region and users should be aware of this possibility.

## Recommended Values and Computer-Program Verification

### Recommended Values

In Table 8, viscosity values are given along the saturation boundary, calculated from the present proposed correlation between 90 K and 150 K, while in Table 9, viscosity values are given for temperatures between 80 K and 150 K and at selected pressures. Saturation density values for selected temperatures, as well as the density values for the selected temperature and pressure are obtained from the equation of state of Tegeler et al. [28]. The values in the tables are calculated from the given temperatures and densities according to Eqs. 1–6.

**Table 8:** Viscosity values of argon along the saturation boundary, calculated by the present scheme

*Τ* (Κ)	*ρ*_liq_ (kg·m^−3^)	*ρ*_vap_ (kg·m^−3^)	*η*_liq_ (μPa·s)	*η*_vap_ (μPa·s)
90	1378.6	7.4362	240.45	7.2246
100	1313.7	16.859	183.07	8.0043
110	1242.8	33.287	142.48	8.8721
120	1162.8	60.144	111.66	9.9017
130	1068.1	103.56	86.679	11.248
140	943.71	178.86	64.471	13.370
150	680.43	394.50	36.712	20.533

**Table 9:** Viscosity values of argon at selected temperatures and pressures, calculated by the present scheme

*p* (MPa)	*T* (K)	*ρ* (kg·m^−3^)	(μPa·s)
0.1	100	4.9152	8.0810
	150	3.2255	12.095
	200	2.4093	15.889
	400	1.2012	28.642
	600	0.80058	38.804
	800	0.60042	47.571
	1000	0.48034	55.485
	1500	0.32025	73.039
	2000	0.24019	88.656
10	100	1349.4	204.28
	150	964.88	67.573
	200	337.74	23.007
	400	119.43	30.618
	600	78.026	39.869
	800	58.472	48.214
	1000	46.880	55.893
	1500	31.435	73.167
	2000	23.672	88.666
50	100	1448.5	290.45
	150	1234.3	129.45
	200	1023.7	79.043
	400	511.19	43.770
	600	342.22	46.526
	800	261.22	52.428
	1000	212.57	58.820
	1500	146.25	74.560
	2000	111.88	89.378
100	100	1528.5	solid
	150	1363.4	187.48
	200	1213.1	121.19
	400	787.37	61.681
	600	574.15	56.530
	800	454.97	59.113
	1000	378.65	63.713
	1500	269.06	77.260
	2000	209.61	91.054
200	100	1635.8	solid
	150	1510.1	309.68
	200	1398.8	198.36
	400	1065.5	93.995
	600	856.29	76.295
	800	717.53	73.178
	1000	619.24	74.551
	1500	464.37	83.938
	2000	373.04	95.656
500	100	1824.2	solid
	150	1739.3	solid
	200	1663.4	486.65
	400	1425.0	191.05
	600	1253.5	134.40
	800	1123.0	114.78
	1000	1019.9	107.27
	1500	834.82	105.83
	2000	709.96	112.26

### Computer-Program Verification

For checking computer implementations of the correlation, the following points may be used for the given *T*, *ρ* conditions: *T* = 300 K, *ρ* = 0 kg·m^−3^, *η* = 22.6840 μPa·s, *T* = 300 K, *ρ* = 4.0 kg⋅m^−3^, *η* = 22.7334 μPa·s, and *T* = 300 K, *ρ* = 700 kg⋅m^−3^, *η* = 49.3360 μPa·s.

## Conclusions

A new, wide-ranging correlation for the viscosity of argon based on critically evaluated experimental data was presented. This correlation is designed to be used over the range of applicability of the equation of state of Tegeler et al. [28] that extends from the triple-point temperature (83.81 K) to 700 K, at pressures up to 1000 MPa. The estimated uncertainty of the correlation based on comparisons with the best experimental data indicate that the uncertainty for the gas at pressures from zero to 0.1 MPa for temperatures from 202 K to 394 K is 0.076%, within the uncertainty of the experimental data [5]. For temperatures above 394 K, comparisons with the data of Vogel et al. [46] show agreement to within the uncertainty of the experimental data, which Vogel conservatively reported as 0.15- 0.2% with the highest uncertainty at the highest temperature. This represents a significant improvement over the current reference correlation of Lemmon and Jacobsen [7] that has an estimated uncertainty of 0.5% in this region. The estimated uncertainty for moderate pressures from 1 MPa to 100 MPa is 1% for temperatures from roughly 195 K to 300 K, rising to 2% at 175 K. For the high-pressure region, the estimated uncertainty of the correlation is about 2% for temperatures between 175 K and 308 K at pressures from 100 MPa to 606 MPa. For temperatures above 308 K to 700 K at pressures to 5.2 GPa, the equation has an estimated uncertainty of 10%. The estimated uncertainty in the liquid phase at pressures up to 34 MPa is 3%.

## Supplementary Information

Below is the link to the electronic supplementary material.

Supplementary file1 (FLD 47 KB)

Supplementary file2 (PDF 141 KB)

## Data Availability

No datasets were generated or analysed during the current study.
