# Supplementary material for: Reference Correlation of the Viscosity of Argon
Source: Int J Thermophys. 2025 Jul 10;46(9):133. doi: 10.1007/s10765-025-03603-8 (PMC12241276; doi:10.1007/s10765-025-03603-8)
Supplement: Supplementary file 2 — Supplementary file2 (PDF 141 KB) [file 10765_2025_3603_MOESM2_ESM.pdf]

Supplemental Information for Reference Correlation of the Viscosity of Argon

Sofia Sotiriadou,[1] Konstantinos D. Antoniadis,[2] Marc J. Assael,[1] and Marcia L. Huber[3,a)]

[1] *Laboratory of Thermophysical Properties and Environmental Processes,*

Chemical Engineering Department, Aristotle University, Thessaloniki 54636, Greece

[2] *Chemical Engineering Department, University of Western Macedonia, Kozani 50100, Greece*

[3] *Applied Chemicals and Materials Division, National Institute of Standards and Technology,*

325 Broadway, Boulder, CO 80305, USA

Additional Details on the Fitting Procedure

The program Eureqa[1] was used with the error metric "absolute error" selected. The formula building blocks were constant, input variable, addition, subtraction, multiplication and division. No variable preparation options were selected. The data were entered as T_r, ρ_r, and the function F (ρ_r,T_r) as described in the text. The function F was set to zero for all points with density less than 2 mol/l in order to force the function to reproduce the theoretical behavior at low density. Due to this, many points at low density in the primary set were not used in the fitting process. Weights were obtained by a trial-and-error process with the goal of representing the majority of the data to within or near the estimated experimental uncertainty. Accordingly, a subset of the primary data listed in Table 2 in the main manuscript were assigned the following weights:

**1st author**	**wt**	**Notes**	**1st author**	**wt**	**Notes**
Zhou	5	vapor points	Mostert	5	
Zhou	8	liquid points	Diller	8	
Humberg	1		Haynes	10	
Berg	1		Vermesse	3	
Abramson	0.5		Rabinovich	10	
Vogel	20		Gracki	1	
Xiao	20		Boon	7	
Hurly	1		Flynn	7	
Evers	3		Flynn	10	Points with T_r>1.49 and ρ_r>0.8
Wilhelm	10				

In addition, to guide the extrapolation behavior at 2000 K, the following points, estimated from a corresponding states method, were added to the fitting procedure.

T_r	ρ_r	F	**wt**	T_r	ρ_r	F	**wt**
13.2725	0.6965	3.429	50	13.2725	1.8469	8.756	50
13.2725	0.9435	4.202	50	13.2725	1.9481	9.511	50
13.2725	1.1495	4.968	50	13.2725	2.6656	17.273	50
13.2725	1.3255	5.371	50	13.2725	3.1301	25.750	50
13.2725	1.4789	6.491	50	13.2725	3.4813	35.050	50
13.2725	1.6147	7.248	50	13.2725	3.7655	45.109	50
13.2725	1.7365	8.002	50				

[1] EUREQA Formulize v.098.1, Nutonian Inc., Cambridge MA, USA (Nutonian Inc., Cambridge MA, USA). Commercial equipment, instruments, or materials are identified only in order to adequately specify certain procedures. In no case does such identification imply recommendation or endorsement by the National Institute of Standards and technology, no does it imply that the products identified are necessarily the best available for the purpose.
